# Symbiotic soil fungi enhance ecosystem resilience to climate change

**DOI:** 10.1111/gcb.13785

**Published:** 2017-07-11

**Authors:** Laura B. Martínez‐García, Gerlinde B. De Deyn, Francisco I. Pugnaire, David Kothamasi, Marcel G. A. van der Heijden

**Affiliations:** ^1^ Department of Soil Quality Wageningen University 6700 AA Wageningen The Netherlands; ^2^ Estación Experimental de Zonas Áridas Consejo Superior de Investigaciones Científicas 04120 Almería Spain; ^3^ Department of Environmental Studies University of Delhi Delhi 110 007 India; ^4^ Plant‐Soil Interactions Research Division of Agroecology and Environmental Science Agroscope CH – 8046 Zürich Switzerland; ^5^ Department of Evolutionary Biology and Environmental Studies University of Zürich CH – 8057 Zürich Switzerland; ^6^ Plant‐Microbe Interactions Institute of Environmental Biology Faculty of Science Utrecht University Utrecht The Netherlands

**Keywords:** arbuscular mycorrhizal fungi, climate change, nitrogen, nutrient leaching, phosphorus, rainfall regimes

## Abstract

Substantial amounts of nutrients are lost from soils through leaching. These losses can be environmentally damaging, causing groundwater eutrophication and also comprise an economic burden in terms of lost agricultural production. More intense precipitation events caused by climate change will likely aggravate this problem. So far it is unresolved to which extent soil biota can make ecosystems more resilient to climate change and reduce nutrient leaching losses when rainfall intensity increases. In this study, we focused on arbuscular mycorrhizal (AM) fungi, common soil fungi that form symbiotic associations with most land plants and which increase plant nutrient uptake. We hypothesized that AM fungi mitigate nutrient losses following intensive precipitation events (higher amount of precipitation and rain events frequency). To test this, we manipulated the presence of AM fungi in model grassland communities subjected to two rainfall scenarios: moderate and high rainfall intensity. The total amount of nutrients lost through leaching increased substantially with higher rainfall intensity. The presence of AM fungi reduced phosphorus losses by 50% under both rainfall scenarios and nitrogen losses by 40% under high rainfall intensity. Thus, the presence of AM fungi enhanced the nutrient interception ability of soils, and AM fungi reduced the nutrient leaching risk when rainfall intensity increases. These findings are especially relevant in areas with high rainfall intensity (e.g., such as the tropics) and for ecosystems that will experience increased rainfall due to climate change. Overall, this work demonstrates that soil biota such as AM fungi can enhance ecosystem resilience and reduce the negative impact of increased precipitation on nutrient losses.

## 1 INTRODUCTION

The use of mineral fertilizers has strongly increased the flow of nitrogen (N) and phosphorus (P) in agricultural systems worldwide and altered global biogeochemical cycles. A significant part of the N and P contained in the fertilizers is lost from agro‐ecosystems via leaching, causing serious groundwater pollution and eutrophication (Galloway et al., 2003). The amount of nutrients applied in agricultural systems is not expected to decline in the coming decades and models predict a global increase of N and P excess of 23% and 54%, respectively, with a major intensification in developing countries (Bouwman et al., 2013; McIntyre, Herren, Wakhungu, & Watson, 2009). Concurrently, climate change models predict changes in rainfall patterns with increased annual precipitation in some regions, and more intense rainfall events (IPCC, 2007). Higher precipitation is often linked to increased nutrient leaching (Austin & Vitousek, 1998), which aggravates the risk of associated human and environmental health problems as highlighted by the Millennium Ecosystem Assessment (MEA, 2005). It is therefore crucial to reduce nutrient losses via leaching to preserve the environment and protect human health. A number of recent studies suggest that soil biota, including arbuscular mycorrhizal (AM) fungi, enhance nutrient cycling in agro‐ecosystems and reduce leaching losses (Bender & Van der Heijden, 2015; Cavagnaro, Bender, Asghari, & van der Heijden, 2015); however, it is not known if AM fungal ability to decrease nutrient losses is maintained across different precipitation scenarios.

AM fungi form symbiotic associations with two‐thirds of all land plants and are widespread in most terrestrial ecosystems (Smith & Read, 2008). Through producing their extensive hyphal network, AM fungi take up soil nutrients and deliver them to their host plants in return for carbon. Therefore, AM fungi play an important role in nutrient cycling (Hodge, Campbell, & Fitter, 2001; Smith & Smith, 2011). Moreover, AM fungi can improve soil structure and plant water relations (Augè, 2004), increasing soil nutrient interception and availability to plants. Consequently, AM fungi have a direct and indirect impact on the amount of nutrients lost from the soil. Recent work suggests that AM fungi reduce P losses via leaching (Asghari & Cavagnaro, 2011; Asghari, Chittleborough, Smith, & Smith, 2005; Bender et al., 2014; Corkidi et al., 2011; Van der Heijden, 2010). Additionally, AM fungi can assimilate N in different forms (Veresoglou, Chen, & Rillig, 2012) and reduce losses of ammonium (NH_4_^+^) and nitrate (NO_3_^−^) from the ecosystem (Cavagnaro et al., 2015). However, the impact of AM fungi on decreasing mineral N losses is variable (Cavagnaro et al., 2015) and might depend on biotic and abiotic factors.

Global warming influences changes in precipitation regimes, which in turn affect terrestrial ecosystem properties (Weltzin et al., 2003) such as soil nutrient cycling. Climate models predict wetter winters in Northern Europe and drier summers in Southern Europe, with higher frequency of heavy rainfall events (Christensen, Goodess, Harris, & Watkiss, 2011). In Northern European countries, the higher frequency and intensity of rainfalls may result in an increase of leaching rates and hence increase of soil nutrient losses (Austin & Vitousek, 1998). By contrast, Mediterranean countries may accumulate inorganic N during dry periods, resulting in higher NO_3_^−^ loss after intense rain events that follow dry periods (Austin et al., 2004). To buffer against high soil nutrient losses from ecosystems and the associated nutrient pollution of drained water under altered rainfall patterns, we need to develop appropriate management practices. This is why it is important to address which factors contribute to nutrient retention under different precipitation regimes so that appropriate management practices can be developed. Moreover, in humid environments with regular and intensive rain (e.g., the tropics), efficient nutrient cycling and low losses is of key importance for maintaining long‐term ecosystem fertility.

The overall goal of this study was to test whether the management of soil biota can mitigate the negative consequences of climate change and in particular of increased rainfall events. Previous studies on how AM fungi impact nutrient leaching have only used one level of rainfall intensity. The novelty of this study is that we simulated two rainfall scenarios: moderate‐ and high‐intensity rain and simultaneously quantified leaching losses of mineral N and P. An experimental grassland community consisting of forbs, grasses, and legumes with or without AM fungi was subjected to the two rainfall levels. Our main objective was to assess whether the presence of AM fungi buffers against the expected increase in nutrient loss under increased rainfall intensity. Specifically, we hypothesized that: (i) the presence of AM fungi will reduce nutrient losses of mineral N and P under both rain scenarios and (ii) the effects of AM fungi in reducing losses are much stronger under a high‐intensity rainfall scenario.

## 2 MATERIALS AND METHODS

### 2.1 Experimental design and growth conditions

We carried out a greenhouse experiment with model grassland communities with and without AM fungi for 6 months. We used 2 L microcosms (diameter 17 cm), filled with 2300 g of autoclaved (2 hr at 110°C) dune sand. Half of the microcosms were inoculated with AM fungi and received 200 g of AM fungal inoculum prepared by combining three different AM fungal strains, namely DD2, DD3, and DD5. The remaining half of the microcosms received the same amount of autoclaved inocula. These AM fungal strains originate from the same Dutch dune grassland (see below and described in Scheublin & Van der Heijden, 2006). To identify the AM fungal species, DNA of the three AM fungal isolates was amplified with the PCR primers NS31 and AM1 (Helgason, Daniell, Husband, Fitter, & Young, 1998; Simon, Lalonde, & Bruns, 1992) and subsequently sequenced. These fungi resemble *Rhizoglomus intraradices* (previously *Rhizophagus intraradices* or *Glomus intraradices* group) (Sieverding, Da Silva, Berndt, & Oehl, 2015), the most widespread AM fungus in this grassland (Scheublin, Ridgway, Young, & van der Heijden, 2004). Sequences were deposited in the GenBank database under accession numbers DQ377988, DQ377991 and DQ487217. A microbial wash was added to all microcosms to correct for potential differences in microbial communities (Koide & Li, 1989). The microbial wash was prepared by wet‐sieving 1000 g of inoculum through a series of sieves (finest sieve was 10 μm) to collect a final volume of 2 L. Each microcosm received 20 ml of microbial wash and a rhizobial suspension with an optical density of 0.24 prepared from isolate nodules of *Lotus corniculatus* as described in Van der Heijden et al. (2006).

Each microcosm received 20 germinated seedlings of the following plant species: two *Hieracium pilosella* L., two *Plantago lanceolata*, two *Lotus corniculata*, two S*enecio jacobea*, four *Festuca ovina*, four *Poa pratensis*, and four *Holcus lanatus* at a fixed position and a fixed distance from each other. The composition of the experimental plant community approximately resembles the Dutch dune grassland community of the study site (as described in Scheublin et al., 2004; Fig. S1). Seeds of these plant species were surface‐sterilized with 1% sodium hypochlorite (commercial bleach) for 10 min, thoroughly rinsed with sterile water and germinated on autoclaved dune sand and planted at fixed positions. The plant species and mycorrhizal fungal isolates used in the microcosms all co‐occurred in a dune grassland ecosystem (Provinciale Waterleidingsduinen Noord Holland; Egmond Binnen; coordinates: 521400N, 041390E), which is referred to as the field site. Dune sand was also collected from this site. In the Netherlands, it is common practice to extract ground water from the dunes as drinking water, such as this field side. Commercially available seeds of wild plants that originated from natural populations in the Netherlands were used (Cruydt‐hoeck, Groningen, the Netherlands).

Plants were grown in a climate‐controlled glasshouse and watered regularly, at a constant temperature of 25°C and a day/night cycle of 10/14 hr. Soil water content in microcosms varied between 13% and 15% for the first 30 days of the experiment. After 30 days, microcosms were subjected to two rainfall regimes, a moderate‐intensity rain treatment and a high‐intensity rain treatment. Soil moisture was controlled by weighing microcosms twice a week and before simulated rainfalls to maintain soil moisture between 13% and 15% for the moderate‐intensity rain treatment and between 19% and 21% for the high‐intensity rain treatment (this treatment regularly received a simulated rain—see below). After 13 weeks of growth, plants were clipped 2 cm above ground. The second growing period lasted also 13 weeks and after a total of 26 weeks, above‐ and belowground plant biomass were harvested. Plants were periodically supplied (approximately once each 2 weeks) with a nutrient solution based on Hoagland's (Table S1; Hoagland & Arnon, 1950). Each microcosm received 12 ml of nutrient solution each time (168 ml total), equivalent to 16.57 kg N/ha and 2.29 kg P/ha.

Overall, the experiment followed a full factorial design, with two factors of two levels each: (i) “AM” (presence and absence of AM fungi) and (ii) “Rain” (moderate and high rain intensity). Therefore, there were four treatment combinations in total, replicated 10 times each. Each microcosm represented an experimental unit and the total number of microcosms was 40.

### 2.2 Simulated rainfall and leachate analysis

Simulated rainfall treatments that varied in precipitation amount and frequency of rain events were applied to all microcosms. Moderate rain intensity treatments received one simulated rainfall at the end of each growing period before harvesting (at week 13 and 26), while the high‐intensity rain treatment received a total of 12 simulated rainfalls (weekly during the first growing period and monthly during the second growing period). Rainfall was simulated by using an acrylic cylindrical container 2 L in volume and 14 cm in diameter fitted with 12 capillars of pore diameter (0.5 mm) at its base and provided with a tripod stand following Knacker et al. (2004). Containers were filled up with 500 ml deionized water during the first six rain events and with 700 ml for the last six rain events in the high‐intensity rain treatment. A precipitation event of 500 ml corresponded to 22 mm rainfall and 700 ml precipitation event corresponded to 31 mm rainfall. Microcosms were placed under the cylinder on plastic plates containing gutters circling toward a central nozzle. A rubber tube fitted to this nozzle channeled the leachate into collection bottles (Fig. S2). After each simulated rainfall, the soil moisture was approximately 23%.

Leachates from each microcosm were collected and 2 hr after the simulated rainfall the bottles were weighed to calculate leachate volume. A subsample of 40 ml for each bottle was stored at −20°C for determination of mineral N (NH_4_^+^‐N, NO_3_^−^‐N) and mineral P (PO_4_^3−^‐P) concentration using a continuous flow autoanalyzer (SKALAR‐SA‐40; Skalar Analytical B.V., Breda, the Netherlands). Mineral N and P losses were calculated by multiplying the concentration of NH_4_^+^‐N, NO_3_^−^‐N and PO_4_^3−^‐P by the volume of leachate.

### 2.3 Plant biomass

Plants were harvested at the end of each growing period. In the first harvest, shoot biomass was clipped 2 cm above soil surface and in the second period, shoot biomass was harvested at soil surface. Shoot biomass was sorted by species. Sand was removed and roots were washed out of soil. Fresh roots biomass was weighed and fresh shoot and root plant mass were dried at 70°C for 3 days and weighed.

### 2.4 Mycorrhizal colonization

To quantify the intensity of AM fungal colonization in plant roots during the experiment, root samples were collected at the end of the first and second growth period. At the end of the first growth period (before harvest), a soil core of 1.5 cm in diameter, 6 cm long was extracted from each microcosm to sample roots and at the end of the second growth period, three subsamples of roots were set aside from the roots collected from each microcosm. Roots were dried at 70°C for 3 days and kept in paper bags until processing. Dried roots were rehydrated, cleared with 10% KOH for 15 min, washed and subsequently stained with trypan blue for 10 min, both in a water bath at 90°C (Phillips & Hayman, 1970). A modification of the line intersection method (McGonigle, Miller, Evans, Fairchild, & Swan, 1990) was used to determine the percentage of root length colonized by AM fungi. One hundred line intersections were scored at each sample for the presence of hyphae, vesicles, and arbuscules.

### 2.5 Statistical analyses

Differences in nutrient losses and total leachate volume among AM fungal and rain intensity treatments were calculated by generalized least square models using “gls” function from the “nlme” package (Pinheiro, Bates, Debroy, & Sarkar, 2014). Mineral N was the sum of NH_4_^+^ ‐N and NO_3_^−^ ‐N losses. Mycorrhizal and rain intensity treatments were treated as categorical factors and plant shoot biomass as a continuous factor to control for its possible influence on leachate properties. The term “weights” was used to account for within‐group heteroskedasticity using “varIdent” function. Pairwise differences were calculated for the interaction between rain and AM treatments using Tukey's post hoc test (significant level set up at 0.05).

Linear mixed‐effects models were used to test AM impact on leachate losses along each growing period within the high‐intensity rain treatment. The “lme” function was used, setting AM treatment as fixed factor and microcosm identity and volume of simulated rainfall (500 ml/week from Rain 1 to Rain 6; 700 ml/week during Rain 7 and Rain 8; 700 ml/month from Rain 9 to Rain 11) as random factors.

Linear models were used to test for the effects of rain and AM treatment on shoot and root biomass and factorial MANOVA was used to test for differences on shoot biomass among the plant functional groups composing the model plant community. Differences of AM colonization (total hyphae, vesicles, and arbuscules) between rain intensity treatments were also tested using linear models. Tukey's post hoc tests were used to check for differences among treatments. Response variables were transformed using logarithm, arcsine, or Box–Cox transformation, when the normality assumption for general lineal models were not achieved.

The relationships between mineral N and P losses and AM fungal colonization (hyphae, vesicles, and arbuscules), total root biomass, and the sum of the shoot biomass from the two growing seasons (grasses, forbs, legumes, and total) were tested separately for each rain treatment using Spearman's correlations.

R software was used for statistical analyses and plot design (R Development Core Team, 2013).

## 3 RESULTS

### 3.1 Effect of AM fungi and rainfall intensity on leaching losses

**Figure 1:**
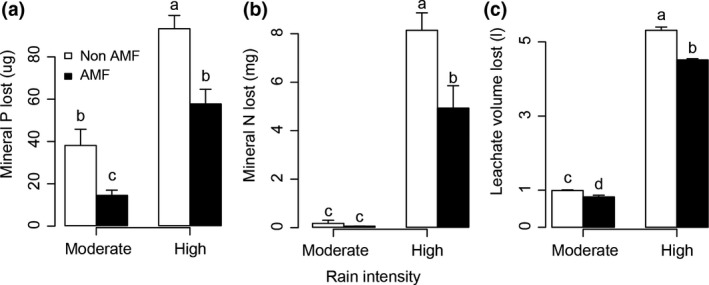
Cumulative mineral P loss (a), N loss (b), and leachate volume losses (c) from microcosms subjected to moderate and high rain intensity treatments in the presence and absence of AM fungi (AMF and non‐AMF, respectively). Bars are means + 1 *SE*; white bars are non‐AMF, solid bars are AMF. Different letters indicate significant difference (*p *< .05) according to Tukey's post hoc test

Rainfall intensity had a strong positive effect on the amount of nutrients lost from soil through leaching (Table 1; Figure 1). Mycorrhizal fungi also influenced the total amount of nutrients lost by decreasing both total mineral P and N loss through leaching (Table 1; F Figure 1a,b). The reduction of P loss from plant communities with AM fungi was approximately the same for both rainfall intensities, around 50%, which corresponds to 0.0133 kg P/ha (Figure 1a). In contrast, the effect of AM fungi on N loss from the plant community through leaching depended on rainfall treatment, (i.e., the interaction between AM and rain treatments was significant [Table 1]). Under high‐intensity rain, AM fungi reduced mineral N loss by almost 40% (corresponding to 1.44 kg N/ha) compared to microcosms without AM fungi, whereas at moderate‐intensity rainfall, there was no significant difference in mineral N loss between treatments with and without AM fungi (Figure 1b). The total volume of leachate differed between AM treatments (Table 1), the volume leached being around 16% lower in presence of AM fungi (Figure 1c).

**Table 1:** F‐values of the lineal models used to test the effect of AM and rain treatments on leachate properties considering the overall experiment, the first growing period and the second growing period. Plant shoot biomass is included as a continuous factor to control for its possible influence on leachate losses

		AM	Rain	AM x Rain	Shoot mass
Overall	*F* (*df*)	*F* (_1, 37_)	*F* (_1, 37_)	*F* (_1, 37_)	*F* (_1, 37_)
	Mineral P	18.62***	63.00***	1.13	1.15
	Mineral N	1.27	111.21***	6.51*	3.48^ms^
	Volume	25.05***	6550.61***	39.72***	0.53
First growing period	*F* (*df*)	*F* (_1, 40_)	*F* (_1, 40_)	*F* (_1, 40_)	*F* (_1, 40_)
	Mineral P	13.510***	22.74***	0.57	0.01
	Mineral N	1.79	108.30***	11.30**	4.41
	Volume	151.58***	7303.23**	92.79***	0.0024
Second growing period	*F* (*df*)	*F* (_1, 37_)	*F* (_1, 37_)	*F* (_1, 37_)	*F* (_1, 37_)
	Mineral P	3.58^ms^	45.78***	0.01	3.52^ms^
	Mineral N	8.73**	607.97***	2.65	0.3
	Volume	0.06	6721.84***	7.77**	5.50*

***<0.001; **<0.01; *<0.05; ms <0.1.

*df*, degrees of freedom.

For both rainfall scenarios, the effect of AM fungi on nutrient losses was tracked after every simulated rainfall across the experimental period (Fig. S3). The presence of AM fungi consistently decreased mineral P losses for both rain treatments (Fig. S3). However, the impact of AM fungi on N loss varied throughout the experiment: in the first growth period mycorrhizal fungi decreased N loss, whereas the effect was inversed during the second growth period, where the presence of AM fungi stimulates total mineral N loss (Fig. S3, Table 1). The later was probably due to N‐release from N‐fixing legumes, which were much more abundant in the treatment with AM fungi (see the next section).

Information regarding the concentration of NH_4_^+^‐N and NO_3_^−^‐N and PO_4_^3−^‐P in the leachate can be found in Table S2.

### 3.2 Effect of AM fungi and rainfall intensity on plant productivity and plant community composition

**Figure 2:**
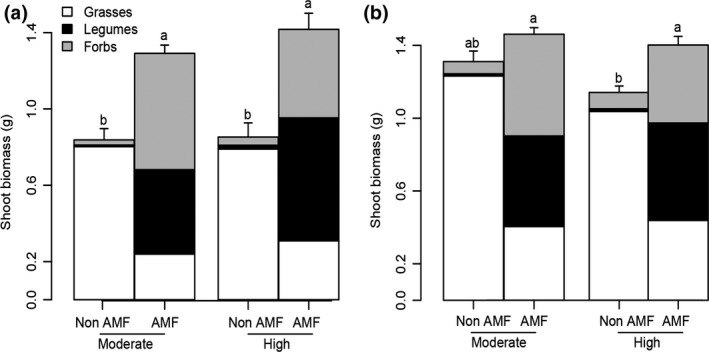
Total mean shoot biomass and standard errors and mean shoot biomass of the plant functional groups (grasses, legumes, and forbs) after (a) first and (b) second growing periods. AMF and non‐AMF indicate presence and absence of AM fungi in microcosms subjected to moderate and high rain intensity treatments. Treatments indicated with different letters are significantly different (Tukey's post hoc test; *p *< .05)

The presence of AM fungi increased total shoot biomass in both growing periods (first growing period; *F*_(1, 40)_ = 49.94, *p <* .001 and second growing period; *F*_(1, 37)_ = 20.55, *p <* .001; Figure 2a,b). AM fungi also had a significant effect on plant species composition and abundance of the three functional groups, i.e., forbs (Hieracium, Plantago, Senecio), legumes (Lotus), and grasses (Festuca, Poa and Holcus) (Table S3). Shoot biomass of forbs and legumes increased in microcosms with AM fungi, whereas grasses dominated microcosms without AM fungi (Figure 2a,b). Root biomass was influenced by rainfall regime, with higher biomass under moderate‐intensity rain than under high‐intensity rain (moderate; 5.25 g ± 0.44, high; 3.94 g ± 0.27; *F*_(1,37)_ = 9.14, *p* < .01; Fig. S4). The presence of AM fungi did not significantly influence root biomass (Fig. S4).

### 3.3 Effects of rain intensity on arbuscular mycorrhizal root colonization

Non‐AM treatments remained free of AM fungal colonization, while colonization of roots in microcosms with AM fungi was successful. Percentage of root length colonized by AM hyphae, vesicles, and arbuscules at the end of the first growing period was not affected by the rain regime (Table 2), while in the second harvest vesicles, arbuscules, and total AM fungal colonization were higher in the moderate than in the high‐intensity rain treatment (Table 2).

**Table 2:** Percentage of AM fungal root length colonization (hyphae, vesicles, and arbuscules) (mean ± 1 *SE*) at the end of the 1st and 2nd growing periods in microcosms subjected to moderate and high rain intensity. Different superscripts letters (a and b) indicate significant difference (*p* < .05) on hyphae, vesicles, and arbuscules between rain regimes (Tukey's post hoc test; *p *< .05)

	First growing period			Second growing period		
	*F*(_1, 20_)	Moderate	High	*F*(_1, 20_)	Moderate	High
Hyphae	2.73	78.30 ± 2.18	73.27 ± 2.17	16.17***	84.33 ± 2.04^a^	70.92 ± 2.76^b^
Vesicles	0.02	44.00 ± 2.30	43.44 ± 2.88	7.57**	48.73 ± 2.81^a^	38.45 ± 2.68^b^
Arbuscules	0.77	8.70 ± 1.45	10.41 ± 1.30	5.48*	14.12 ± 1.91^a^	9.11 ± 1.17^b^

***<0.001; **<0.01; *<0.05.

### 3.4 Relationship between AM fungal colonization, plant biomass, and nutrient leaching losses

Phosphorus losses decreased with higher AM fungal colonization in both rain regimes. Specifically, total hyphal colonization and vesicles (i.e., assumed to be a nutrient reservoir organelle) negatively correlated with P losses for both rain treatments, while arbuscules percentage (i.e., assumed to be a nutrient transfer organelle) only correlated to P losses within the moderate‐intensity rain treatment (Table 3). Similarly, N losses decreased with higher AM fungal colonization (hyphae, vesicles, and arbuscules) within the high treatment but not within the moderate‐intensity rain treatment (Table 3).

**Table 3:** Spearman's correlation coefficients between total N and P losses, AM fungal colonization (hyphae, vesicles, and arbuscules) and plant biomass (shoot, roots, grasses, forbs, and legumes) using data of moderate and high rain intensity scenarios. Correlations are performed first including AMF and non‐AMF treatments (*n* = 40) and then, including only the AMF treatments (*n* = 20)

		AMF and Non‐AMF treatments				Within AMF treatment			
		Moderate rain		High rain		Moderate rain		High rain	
		N loss	P loss	N loss	P loss	N loss	P loss	N loss	P loss
AM fungal colonization	Hyphae	−0.11	−0.58**	−0.53*	−0.56**	0.38	0.2	0.03	−0.02
	Vesicles	0.12	−0.58**	−0.61**	−0.49*	0.38	0.2	−0.32	0.15
	Arbuscules	−0.23	−0.55*	−0.61**	0.43	0.09	0.44	−0.44	0.47
Plant biomass	Root	0.22	0.51*	−0.46*	−0.2	0.16	0.31	−0.27	0.07
	Shoot	−0.55*	−0.63**	−0.64**	−0.4	−0.59	−0.47	−0.58	0.05
	Grasses (shoot)	0.03	0.55*	0.50*	0.43	−0.19	−0.43	−0.01	−0.45
	Forbs (shoot)	−0.32	−0.62**	−0.58**	−0.48*	−0.16	0.18	−0.3	0.09
	Legumes (shoot)	−0.27	−0.61**	−0.58**	−0.51*	−0.03	−0.05	−0.49	−0.07

**<0.01; *<0.05.

Correlations between total plant biomass and nutrient losses suggest that N losses were more strongly related to plant biomass than P losses. Larger shoot biomass consistently showed lower N losses under both rain regimes, whereas P losses only related negatively to shoot biomass within the moderate rain treatment (Table 3). Additionally, N loss through leaching also declined with larger root biomass within the high rain intensity treatment (Table 3).

Induced changes in plant community composition through altered biomass of the different plant species in response to the presence of AM fungi were correlated to the amount of nutrients lost via leaching. Likewise, the biomass of forbs and legumes (plant functional groups enhanced by AM fungal presence) was negatively correlated with nutrient losses (N and P) under high rain regime. In contrast, the biomass of grasses was positively correlated to nutrient losses, enhancing P loss in the moderate rain treatment and N loss in the high‐intensity rain treatment (Table 3).

The results in Table 3 include data from all microcosms including mycorrhizal and nonmycorrhizal treatments. In a next step, we removed the data from nonmycorrhizal microcosms and tested whether differences in root colonization could explain differences in nutrient loss across the mycorrhizal microcosms. No significant correlations were observed (Table 3). These results indicate that especially differences between mycorrhizal and nonmycorrhizal microcosms are important in explaining nutrient losses. However, the variation of the level of colonization was small between the replicates of the inoculated communities (it varied from 74% to 94% and from 62% to 80% within the moderate and high rain treatment, respectively), compared with the variation of AM fungal root colonization among natural grasslands. A more appropriate way to specifically test the effect of differences in fungal colonization would have been to test a gradient of mycorrhizal abundance created as independent variable (e.g., by using different inoculum densities).

## 4 DISCUSSION

Our data show that AM fungi can decrease rain‐induced nutrient losses by leaching. Moreover, the extent to which AM fungi contribute to reduced mineral N loss depends on rainfall intensity; the presence of AM fungi had the biggest contribution to the reduction of mineral N loss when rainfall intensity was high. We also confirmed that the beneficial effect of AM fungi at reducing mineral P loss via leaching (Asghari et al., 2005; Corkidi et al., 2011; Van der Heijden, 2010) is maintained under different rain intensity scenarios. This highlights the importance of AM fungi for sustainable nutrient cycling and thus, for ecosystem resilience. This is especially relevant in areas with high rainfall intensity (such as the tropics) where high precipitation rates will cause major nutrient losses via leaching. In addition, this finding is important for ecosystems that will experience increased rainfall due to climate change. While earlier studies reported that AM fungi can reduce nutrient leaching losses from agricultural and natural settings (e.g., Cavagnaro et al., 2015), this study shows that this function of AM fungi is maintained, and even improved, when rainfall intensity is higher.

The AM fungal symbiosis provided additional benefits to the plant community. For instance, the presence of AM fungi enhanced shoot biomass, plant community evenness and plant diversity. High plant community evenness is essential to preserve grasslands multifunctionality (Hector and Bagchi, 2007), including increased resilience of grassland ecosystems to changing climate conditions (Isbell et al., 2015). AM fungi enhanced growth of forbs and legumes and changed plant community structure from grass‐dominated systems to others where grasses, forbs, and legumes had similar abundance. Such shifts are highly relevant in the context of nutrient leaching, as earlier studies showed that differences in the total biomass of the plant community and in the relative abundance of the plant functional groups can influence the amount of nutrient loss in grassland systems (De Deyn et al., 2009; Oelmann et al., 2007; Scherer‐Lorenzen, Palmborg, Prinz, & Schulze, 2003).

Below we discuss the main mechanisms that drive nutrient losses (mineral N and P) under the two rain intensity scenarios. The decrease in P losses was probably caused by the ability of AM fungi to take up soil P. It is well known that the major advantage of AM symbiosis for plants is the increase of P uptake, especially in systems where soil P concentration is low, as in our soil substrate (Smith & Read, 2008). In our experiment, AM fungi halved the amount of mineral P loss via leaching under both rain regimes (Figure 1) and this effect was maintained across the experiment. Moreover, mineral P losses decreased with higher mycorrhizal colonization for both moderate and high rain intensity scenarios (Table 3). Maintaining the natural mycorrhizal fungal networks is therefore essential for a sustainable P management, as higher P uptake will result in lower concentrations of the soil phosphates to be leached with rain events, and consequently, lower risk of groundwater contamination. The effect of AM fungi on P losses is especially relevant for sandy soils (such as in this study) or other highly permeable soils where P loss can be substantial (Sims, Simard, & Joern, 1998). In other soils, P leaching losses are often very low and P loss through surface run‐off is much more important (Sharpley, Mcdowell, & Kleinman, 2001). Moreover, currently available P resources are becoming increasingly scarce (Cordell & White, 2014); therefore, it is crucial to adapt actual land management practices to natural strategies that enhance nutrient use efficiency.

The ability of AM fungi to decrease mineral N losses was clear in the high rain intensity scenario, where AM fungi reduced total N loss by 40% (from 3.66 kg N/ha to 2.22 kg N/ ha in nonmycorrhizal to mycorrhizal microcosms, respectively; Figure 1). Accordingly, AM fungal colonization negatively correlated with the amount of N lost in the high‐intensity rain regime (Table 3). This result supports the recently shown ability of AM fungi to decrease N losses (Asghari & Cavagnaro, 2012; Bender & Van der Heijden, 2015) and highlights the relevance of AM fungi to retain soil nutrients especially in ecosystems with higher precipitation rates. At the moderate rain intensity scenario, we did not find an effect of the presence of AM fungi on N loss via leachate; however, under this rain regime, the amount of N loss was very small (0.06 kg N/ha). The potential of AM fungi to retain nutrients may be much higher in systems with high N losses (e.g., in some agricultural areas up to an estimated 160 kg N/ha year is lost through leaching [Bender & Van der Heijden, 2015; Herzog, Prasuhn, Spiess, & Richner, 2008], much more compared to values observed in this experiment). Here we focused on N leaching losses, while we did not measure gaseous N losses through the process of denitrification (e.g., Bender et al., 2014; Cavagnaro, Barrios‐Masias, & Jackson, 2012; Seitzinger et al., 2006).

Our results also suggest that AM fungi indirectly influenced N losses by the shift in plant community structure (Figure 2). For instance, it may surprise that with increased abundance of legumes and decreased abundance of grasses in the presence of AM fungi, leaching of N is reduced specially at the high rain intensity treatment, given earlier reports of increased N loss with increased abundance of legumes (Scherer‐Lorenzen et al., 2003). However, in our experiment also the abundance of forbs increased with AM fungi as well as total biomass, which can all counteract leaching losses (De Deyn et al., 2009). Moreover during plant growth, legumes take up and immobilize N in their tissues; therefore, their impact on N leaching may initially be negative but changes over time as their N‐rich roots start to turn over and releases N.

The role played by AM fungi in decreasing N loss was crucial in the first rain event, when 80% of the total N loss during the experiment was leached (Fig. S3). Microcosms with presence of AM fungi had 50% less N loss, which corresponds to a decrease of 1.34 kg N/ha after a single rain event. The difference in the amount of N leached between mycorrhizal and nonmycorrhizal microcosms at the first rain (week 6), is likely due to the presence of a mycorrhizal mycelium that extends into the soil matrix and acquires soil nutrients present in the fine pores that are not accessible to roots. The pots were only slightly fertilized during this experiment and the bigger amount of N loss during the first rain (2.99 kg/ha in nonmycorrhizal microcosms and 1.65 kg/ha in mycorrhizal microcosms) compared to the consecutive 11 simulated rains (0.054 kg/ha average) was probably due to high initial soil nutrient availability and the absence of a well‐developed root system. This result also shows that the presence of AM fungi enhances the nutrient interception ability of soils and decreases the nutrient leaching risk by enhancing nutrient uptake and immobilizing nutrients (Bender, Wagg, & Van der Heijden, 2016).

In addition to reducing nutrient loss, AM fungi significantly reduced the volume of leachate. The higher water uptake by plants via mycorrhizal mycelium and increased plant transpiration because of higher plant biomass in mycorrhizal treatments could explain the reduced leaching volume. Moreover, it is known that fungal hyphae improve soil structure by stabilizing soil micro‐ and macro‐aggregates (Miller & Jastrow, 2000; Rillig & Mummey, 2006) and this is another way that can affect soil water relations and leaching volume. For instance, Augè (2004) showed that AM fungi improve soil moisture retention in a sandy soil. In this experiment, we did not measure whether AM fungi induce changes in soil structure via mycorrhizal mycelium; nevertheless, the mycorrhizal mycelium may contribute to enhance water retention. This effect may be more relevant in sandy systems, like in our field site, where the risk of nutrient leaching is high, and the groundwater from such system is used as a source of human drinking water (Piet & Zoeteman, 1980).

Changes in rainfall patterns will modify the soil water dynamics and related ecosystem processes such as N cycling that is intimately linked to soil moisture. It is expected that increased precipitation will enhance the number of wet–dry cycles and increase nitrification and denitrification processes (Knapp et al., 2008). For instance, higher precipitation intensity enhances the period of time that the soil is saturated with water and therefore denitrification increases. In our experiment, anaerobic conditions were more frequent in the high rain intensity treatment as it received 12 simulated rainfalls, while the moderate rain intensity treatment only received two simulated rainfalls. Still, we expect that the anaerobic conditions were of short duration as soil moisture after the rain events quickly dropped due to the texture of the substrate used (dune sand is mainly composed by large sand particles with hardly any clary or silt). When oxygen levels are not limiting, higher soil moisture enhances ecosystem activity such as plant nutrients uptake and N mineralization. Therefore, we expect that under water excess there will be higher N losses both via gaseous emissions (NO_3_ to N_2_O) and via leaching when N availability exceeds N‐uptake. Our results together with previous studies that have shown that AM fungi can decrease N_2_O fluxes (Bender et al., 2014) especially under moist conditions (Lazcano, Barrios‐Masias, & Jackson, 2014), highlight the importance of AM fungi to prevent N loss in wet ecosystems.

There are limitations in using results from controlled greenhouse experiments to predict nutrient losses in field conditions. We chose to use microcosms with sterilized sand and AM fungal inoculation (together with the addition of microbial communities) to successfully detach the effect of mycorrhizal fungi on leachate properties. Our microcosms resembled Dutch dune grasslands with similar plant community and vegetation density and AM fungal strains that were isolated from the field site. However, the results do not mirror real conditions as there is absence of soil macrofauna, limited rooting depth, and lower number of AM fungal taxa compared to natural communities. The AM fungal strains used are indigenous from the field site, and therefore the ecosystem functions that are provided by decreasing nutrient leaching and maintaining plant diversity can be also attributed to the mycorrhizal communities in the field site. However, it is important to consider that different fungal taxa influence plant nutrient uptake and nutrient leaching properties differently (Köhl & Van der Heijden, 2016; Martínez‐García, Ochoa‐Hueso, Manrique, & Pugnaire, 2015) and that lower mycorrhizal diversity compared to natural ecosystems may decrease AM fungal multifunctionality; therefore, our results cannot be applied generally to every ecosystem. Moreover, the rainfall scenarios used in this experiment are not representative of climatic predictive models but simulate a general intensification of rainfall based on higher precipitation amount and higher frequency of rainfall events than in the region of our study system. Further field studies assessing how real climate change scenarios impact on ecosystem properties (such as in Martínez‐García, De Dios, & Pugnaire, 2012) are also needed to predict accurately nutrient losses in ecosystems.

Overall, our study demonstrates that the mycorrhizal symbiosis in grasslands contribute to multiple ecosystem services such as reducing nutrient loss, improving nutrient cycling, and enhancing plant diversity. As a result, AM fungi enhance ecosystem resilience to changes in precipitation patterns. These findings are of special relevance in habitats were change in rainfall patterns will increase the amount of nutrients lost via leaching. For instance, in agricultural grasslands where there is a large input of N and P through fertilizers or in sandy soils where nutrients rapidly leach after rain events. By reducing N and P loss in the leachate, AM fungi improve nutrient cycling and prevent groundwater nutrient leaching pollution. Similarly, by increasing plant functional diversity and enhancing the abundance of legumes and forbs, AM fungi preserve grasslands multifunctionality (Hector and Bagchi, 2007). Implementation of management practices that maintain natural biotic interactions (such as the AM–plant symbiosis) is essential to maintain the resilience of ecosystems to climate change.

## CONFLICT OF INTEREST

Authors do not declare any conflict of interest or relationship that might influence author's objectivity.
