# Supplementary material for: Symbiotic soil fungi enhance ecosystem resilience to climate change
Source: Glob Chang Biol. 2017 Jul 11;23(12):5228–36. doi: 10.1111/gcb.13785 (PMC5697572; doi:10.1111/gcb.13785)
Supplement: Supplementary file 1 [file GCB-23-5228-s001.pdf]

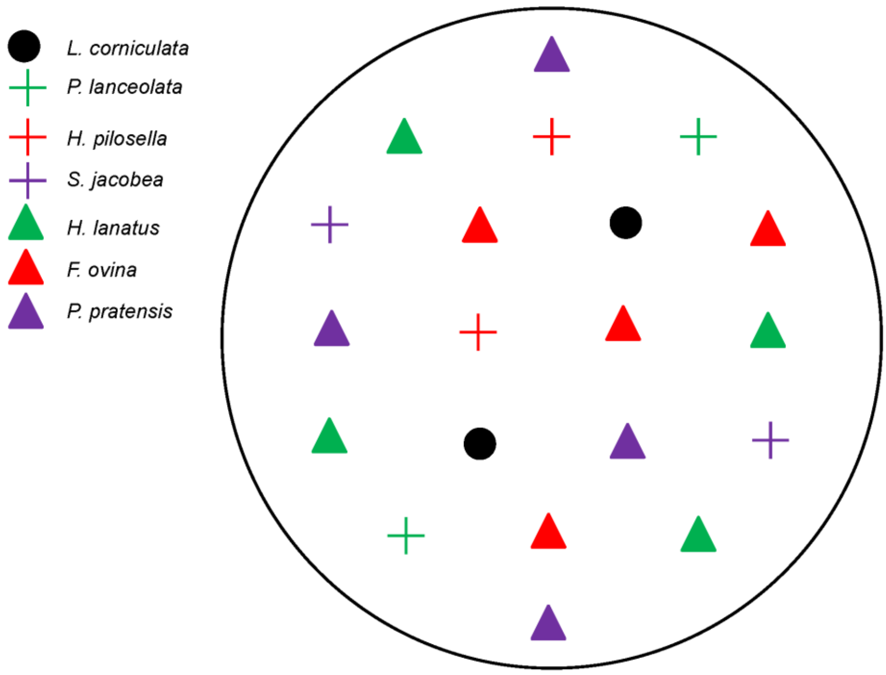

Fig. S1. The location of plant species (and number of individuals per species) in the microcosms.

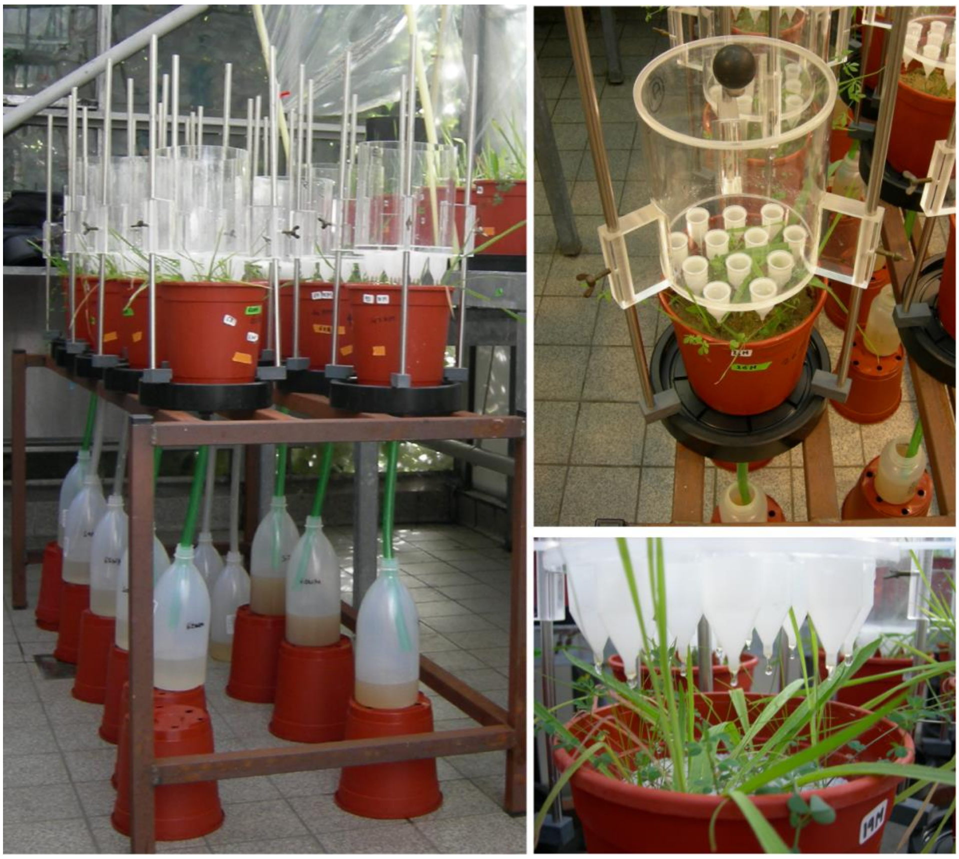

Fig. S2. Rainfall simulator device.

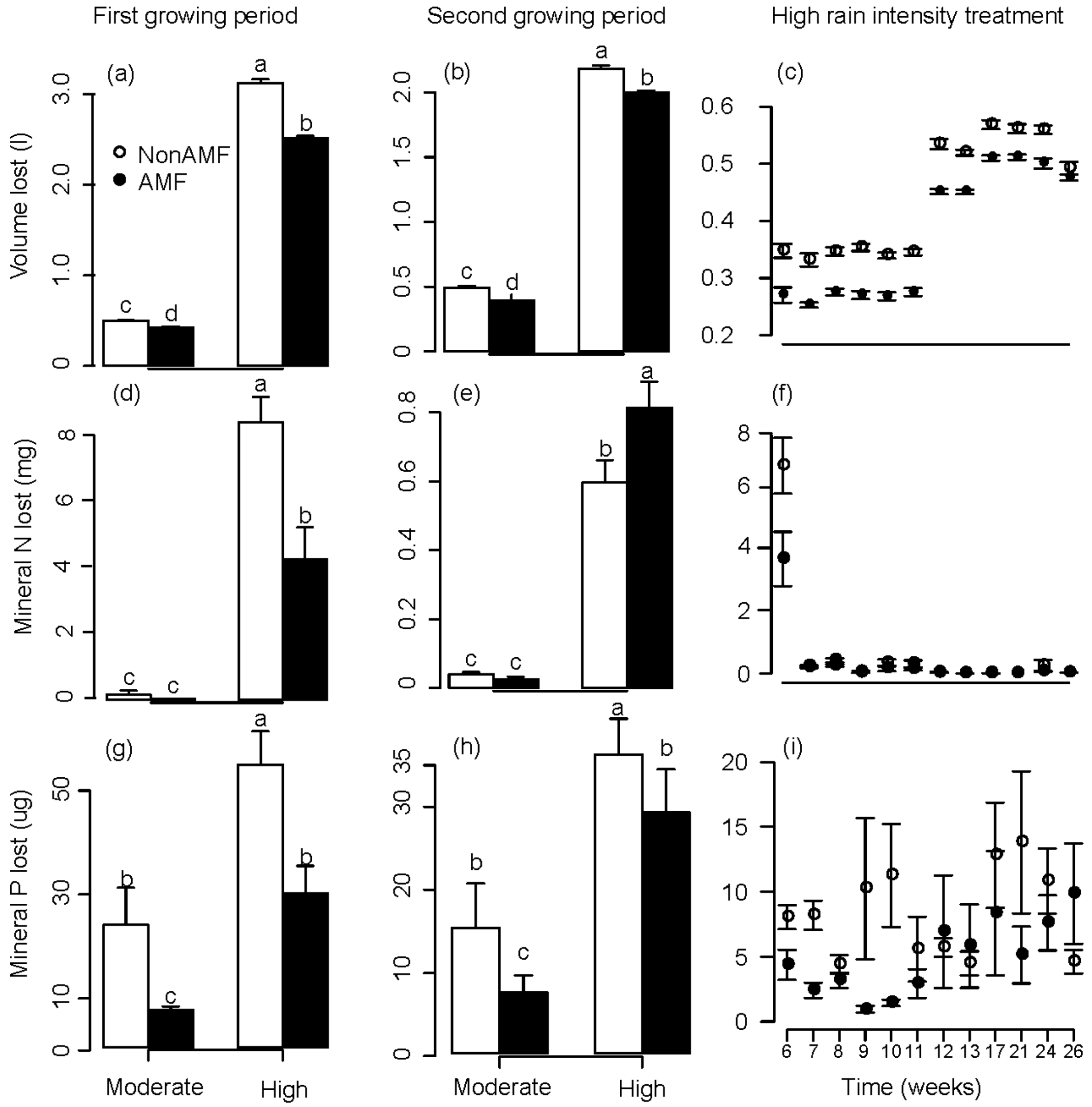

Fig. S3. Leachate volume (a, b, c), mineral N (d, e, f) and P losses (g, h, i) during the first growing period, the second growing period and at each simulated rainfall respectively, from microcosms subjected to moderate and high rain intensity treatments. Bars are means + 1SE, white bars and circles indicate absence of AM fungi (AMF) and dark bars indicate presence of AMF. Different letters indicate significance difference (*P* < 0.05) according to Tukey's post-hoc test.

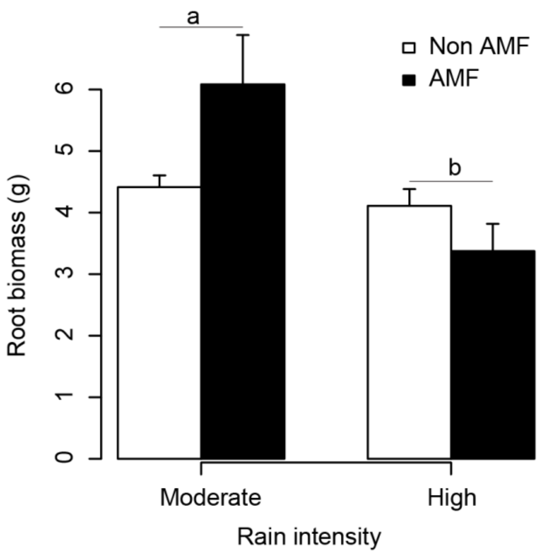

Fig S4. Mean root biomass and standard errors at the end of the experiment in microcosms with (black bars) or without AMF (white bars) and at Moderate or High rain intensity treatments. Different letters indicate significant difference between moderate and high rain intensity treatments according to Tukey's post-hoc test ($P < 0.05$).

Table S1. Adapted Hoagland nutrient solution. Each microcosms received 12 ml of this solution approximately each 2 weeks. The cumulative added solution was 168 ml which corresponds to 16.57 kg N/ha and 2.29 kg P/ha.

**Hoagland Nutrient Solution**	
**Macronutrients**	mmol/l
KNO_3	6
$CaCl_2$	4
$Ca(NO_3)_2.4H_20$	0.5
NH_4NO_3	0.5
$NH_4H_2PO_4$	1
$MgSO_4.6H_20$	1
**Micronutrients**	
KCL, H_3BO_3, $MnSO_4.4H_20$, $ZnSO_4.4H_20$, $CuSO_4.4H_20$, $(NH_4)6Mo_7O_{24}.4H_20$	1
Fe(Na)EDTA, $C_{10}H_{12}N_2O_8FeNa$	1

Table S2. NH_4^+-N and NO_3^--N and PO_4^{3-}-P concentration in the leachate collected after each induced simulated rainfall (mean ± 1 SE) from microcosms subjected to moderate and high rain intensity treatments, in presence and absence of AM fungi (AMF and Non AMF respectively).

		Moderate		High	
**Week**	**Rain**	**Non AMF**	**AMF**	**Non AMF**	**AMF**
			**NH_4^+-N (mg/l)**		
**w-6**	**R-1**			0.2461 ± 0.0652	0.0605 ± 0.0041
**w-7**	**R-2**			0.0752 ± 0.0277	0.0549 ± 0.0054
**w-8**	**R-3**			0.026 ± 0.0074	0.0283 ± 0.0115
**w-9**	**R-4**			0.0173 ± 0.0024	0.0141 ± 0.0018
**w-10**	**R-5**			0.0138 ± 0.0043	0.009 ± 0.0021
**w-11**	**R-6**			0.0098 ± 0.0013	0.0094 ± 8e-04
**w-12**	**R-7**			0.0105 ± 0.0034	0.0056 ± 0.0017
**w-13**	**R-8**	0.0379 ± 0.0022	0.0303 ± 0.0037	0.0196 ± 0.0041	0.0275 ± 0.005
**w-17**	**R-9**			0.0331 ± 0.0036	0.0534 ± 0.0106
**w-21**	**R-10**			0.0316 ± 0.0032	0.0282 ± 0.0049
**w-24**	**R-11**			0.0256 ± 0.0025	0.0196 ± 0.0019
**w-26**	**R-12**	0.0787 ± 0.0142	0.0468 ± 0.0112	0.0516 ± 0.0217	0.0379 ± 0.0091
			**NO_3^--N (mg/l)**		
**w-6**	**R-1**			20.9564 ± 7.2348	13.6751 ± 12.8766
**w-7**	**R-2**			1.1062 ± 1.7742	0.6238 ± 1.3905
**w-8**	**R-3**			0.9572 ± 1.5892	0.5158 ± 0.6871
**w-9**	**R-4**			0.1111 ± 0.1498	0.0874 ± 0.0865
**w-10**	**R-5**			0.0123 ± 0.0334	0.0059 ± 0.0111
**w-11**	**R-6**			0.0137 ± 0.0331	0.0311 ± 0.0602
**w-12**	**R-7**			0.0095 ± 0.0216	0.0088 ± 0.0099
**w-13**	**R-8**	0.1549 ± 0.558	0.0586 ± 0.0638	0.4848 ± 1.5405	0.1624 ± 0.1711
**w-17**	**R-9**			0.0423 ± 0.03	0.0439 ± 0.0276
**w-21**	**R-10**			0.3586 ± 0.1881	0.4671 ± 0.245
**w-24**	**R-11**			0.5153 ± 0.3695	0.8675 ± 0.6342
**w-26**	**R-12**	0.0065 ± 0.0114	0.0209 ± 0.0349	0.0365 ± 0.0351	0.1066 ± 0.3245
			**PO_4^{3-}-P (ug/l)**		
**w-6**	**R-1**			21.4197 ± 5.7013	15.9823 ± 8.7691
**w-7**	**R-2**			23.4345 ± 9.1532	11.0979 ± 5.6804
**w-8**	**R-3**			13.1597 ± 5.9983	12.6919 ± 5.6649
**w-9**	**R-4**			26.3315 ± 39.3894	5.9842 ± 2.2786
**w-10**	**R-5**			29.561 ± 27.5722	7.5783 ± 2.6661
**w-11**	**R-6**			15.8685 ± 18.5179	12.1805 ± 11.5426
**w-12**	**R-7**			10.4564 ± 3.4966	14.2385 ± 23.4253
**w-13**	**R-8**	42.4893 ± 41.1669	17.2849 ± 5.2032	8.8062 ± 4.7686	12.6686 ± 18.6603
**w-17**	**R-9**			20.0592 ± 18.0971	15.3349 ± 24.3487
**w-21**	**R-10**			21.7166 ± 23.847	9.7488 ± 10.4305
**w-24**	**R-11**			17.5442 ± 11.429	14.3647 ± 10.9194
**w-26**	**R-12**	31.629 ± 36.6876	19.2121 ± 14.3934	9.6672 ± 5.282	19.1437 ± 20.8914

Table S3. F-values of the lineal models used to test the effect of arbuscular mycorrhizal fungi (AMF) and rain intensity treatments (Rain) on total shoot biomass and plant functional groups (grasses, legumes and forbs) shoot biomass after the 1st and 2nd growing periods. Significant P values are in bold. Df: degrees of freedom.

		**1st Growing Period**		**2nd Growing Period**	
	**Df**	**F value**	**P**	**F value**	**P**
Total shoot biomass					
AMF	1	40.94	< **0.001**	20.549	< **0.001**
Rain	1	1.98	0.17	6.368	< **0.05**
AMF x Rain	1	0.001	0.97	1.471	0.2331
Residuals	36				
Grasses					
AMF	1	82.32	< **0.001**	240.32	< **0.001**
Rain	1	3.95	0.057	1.92	0.17
AMF x Rain	1	0.03	0.87	5.29	**0.03**
Residuals	27				
Legumes					
AMF	1	260.16	< **0.001**	775.53	< **0.001**
Rain	1	3.06	0.09	1.5	0.23
AMF x Rain	1	0.18	0.68	0.07	0.79
Residuals	27				
Forbs					
AMF	1	172.25	< **0.001**	404.2	< **0.001**
Rain	1	0.59	0.45	1.52	0.22
AMF x Rain	1	2.88	0.1	9.48	< **0.01**
Residuals	27				
